# The bright and dark sides of protein conformational switches and the unifying forces of infections

**DOI:** 10.1038/s42003-020-1115-x

**Published:** 2020-07-15

**Authors:** Achinta Sannigrahi, Nayan De, Krishnananda Chattopadhyay

**Affiliations:** Structural Biology & Bio-Informatics Division, CSIR-Indian Institute of Chemical Biology, 4, Raja S. C. Mullick Road, Kolkata, 700032 India

**Keywords:** Structural biology, Proteins, Protein sequence analyses

## Abstract

It is now established that a protein can switch between multiple conformations to enable altered functions. Several pathogens including SARS COV2 utilize context-dependent conformational switches of particular proteins to invade host membrane to establish infections. In this perspective, we first discuss the understanding of the conformational switch of a protein towards the productive infections as a dark side of nature. Next, the unexplored binary combination of the sequences of SARS COV2 spike protein and the similarity with diverse pathogen derived proteins have been discussed to obtain novel molecular insights into the process of infection.

## Abstract (web summary)

Sannigrahi et al. discuss conformational switching that allows proteins to alter their conformation, aiding in pathogenesis by enabling cellular entry of viruses and bacterial toxins. Focussing on fusion domain of the Spike glycoprotein of SARS COV2 virus, they identify similar “switching sequences” present in diverse pathogen derived proteins.

## Introduction

Understanding the entry process of pathogens (including virus, bacteria, and other parasites) inside hosts is crucial in order to design the strategy of resistance. In this context, it is well known that many pathogenic systems utilize specific protein molecules, which play crucial roles in the internalization process of the pathogens. These pathogen-derived proteins may not show activity in their native states, while undergo inactive to active state conformational transitions when introduced to external stimuli, including pH, surface receptor binding, post-translational modifications, and mutations. This change in conformation from one state to another of potentially varying functions is sometimes referred as conformational switch^1^. The environment-sensitive conformational change of a protein is critical determinant of its biological function^2^. In 1959, a talk by Dr. Richard P. Feynman entitled, “There’s plenty of room at the bottom”, tried to attract the scientific community’s focus toward the possibilities of nanotechnologies. In this talk, he was motivated by some biological systems which play their distinct roles in different contexts and these functions are separated from one another by a small energy barrier. Proteins are the most common examples of these systems that can switch their functions with the change of environmental conditions. Protein designers are now trying to create protein sequences that can adopt numerous specific conformations and to control the relative stability of these states, advancing the molecular machines envisioned by Dr. Feynman.

## Conformational switches in bacterial and parasite proteins

Conformational switches that occur naturally can be of different forms. Some exhibit a change from a disordered to an ordered state, whereby the transition would encompass an entire protein or a portion of a protein. In several other cases, there would be a switch between different ordered conformations. In this connection, a particular conformational state of a protein may be the decisive factor toward a particular disease process.

Many pathogenic bacteria e.g., staphylococcus aureus, corynebacterium diphtheria use pore-forming toxins (PFT), which are considered the principal virulence factors. PFTs are generally utilized by the disease-causing pathogens to disrupt the first line of defence of the host i.e the host cell membranes through pore formation strategy. Two classes of PFTs (α and β) are well documented on the basis of their conformation of the membrane interacting domain^3–5^.

**Fig. 1:**
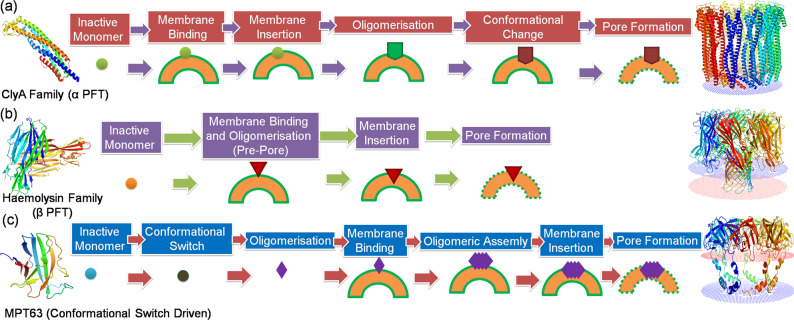
The membrane pore formation mechanism of different kind of PFTs. **a** ClyA family proteins have been mentioned here as an example of α-PFTs. The solved monomeric soluble structure (PDB ID: 1QOY) and the dodecameric structure (PDB ID: 6MRT) of ClyA are shown in the figure along with the mechanism of pore formation. There is no pre-pore structure in case of ClyA. **b** The Haemolysin family proteins are given as an example of β-PFTs. The solved monomeric soluble structure of α-Hemolysin (PDB ID: 2QK7) and the oligomeric structure (PDB ID: 7AHL) are shown in the figure along with the mechanism of pore formation. The pre-pore formation is one of the key mechanistic steps during membrane pore formation by β-PFT family proteins. **c** The mechanism of pore formation by MPT63 (PDB ID: 1LMI) by its conformational switch strategy and the predicted membrane-associated structure (model) has been shown. Here oligomeric assembly is the new mechanistic pathway of pore formation.

Many α PFTs possess native alpha-helical structure and some of the alpha-helical regions get inserted into membrane during interaction (e.g., Colicin A, Cly A family, Fig. 1)^6, 7^. In solution, ClyA remains predominantly alpha-helical except a short hydrophobic beta-hairpin, which is called as the beta tongue, which detaches from the core of the protein and goes inside the lipid bilayer. Subsequently, pore formation by ClyA occurs through oligomerisation through a sequential mechanism. β-PFTs form pores by the insertion of amphipathic beta-hairpin into the membrane to create a beta-barrel (e.g., Staphylococcus aureus alpha-hemolysin (α HL)^8–10^. The mechanism of β−PFTs is based on the nature of the toxins i.e. either single or bi-components. The single-component toxins of this family assemble into heptamaeric pores whereas bi-components toxins form octameric pores. It is believed that pre stem-loop is not required for the oligomerisation process of β-PFT, and is essential for the oligomerisation process in case of α-PFTs (Fig. 1).

**Fig. 2:**
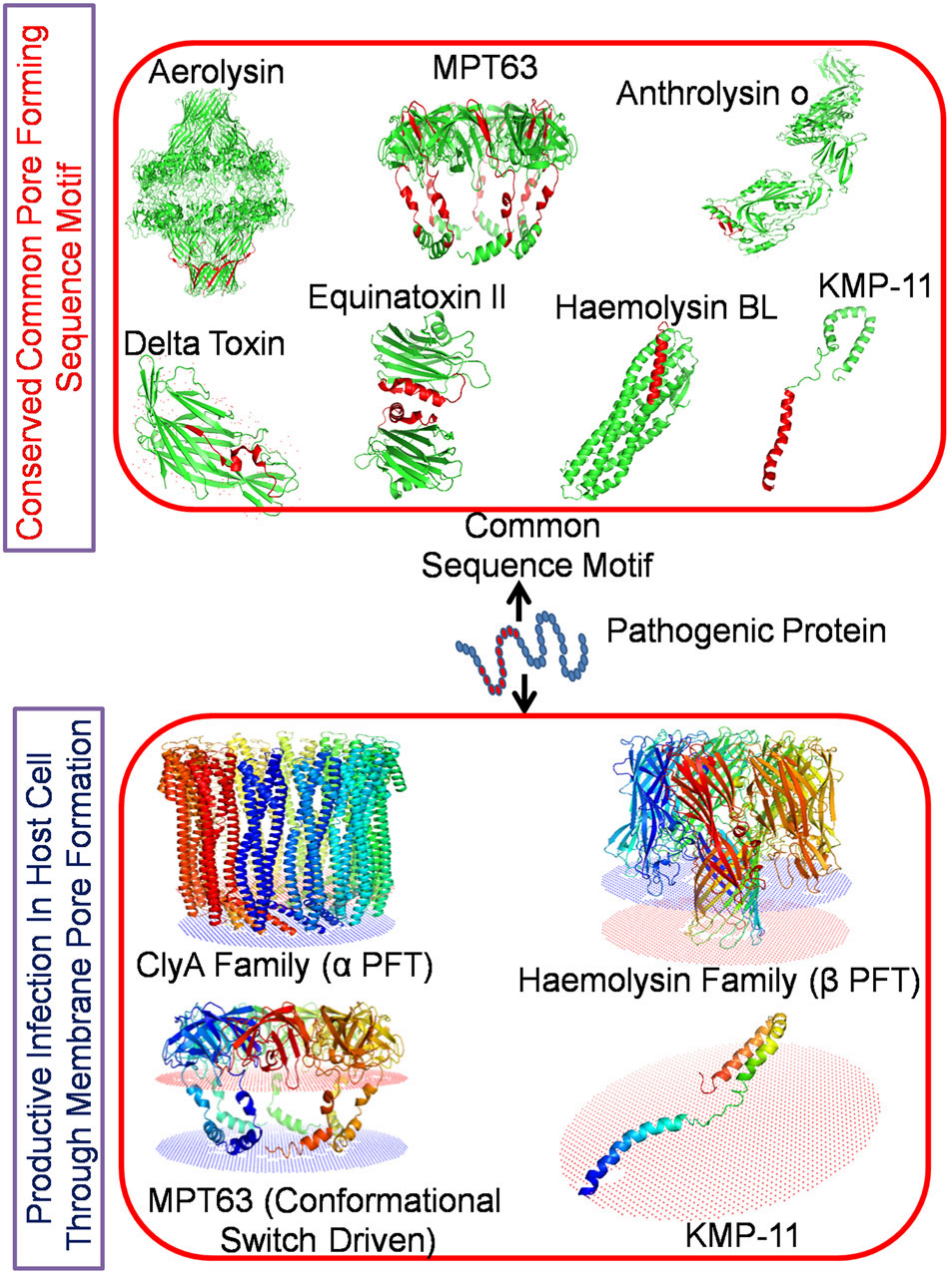
The schematic representation shows the conformational switch of a protein. There is common sequence motif in different membrane pore-forming proteins (PFTs). Pathogen utilizes this conformational switch strategy to evade the host cell membranes. The common motif would be useful for designing appropriate drug candidates, antibody/small molecule/peptide blocker to inhibit various pathogenic infections. The membrane-associated structures were obtained using OPM server (http://opm.phar.umich.edu/).

In addition to the above two, there exists a special class of toxins, which exhibits pore-forming character using conformational switch strategy when triggered by external factors. Mycobacterium tuberculosis is known to modulate host immune responses to facilitate its persistence inside host cells. It has been recently shown that a small immunogenic protein, MPT63 in TB switches from its native predominantly beta-sheet to alpha-helical structure. This alpha-helical form undergoes oligomeristaion to form toxic ‘oval’ shaped morphology at pH 5 (atmosphere inside granulomatous M2 macrophages). These toxic oligomers create pores in macrophages and model membrane. Figure 1 shows oligomerisation and pore formation processes of MPT63, which differ from both α and β PFTs. When alpha-helical MPT63 comes in contact with membrane, it forms an apparent five-unit oligomeric assembly on the membrane resulting membrane rupture, toxicity consequences, and cell death. Interestingly, N-terminal chameleon stretch of MPT63 (19–33 AA) remains conserved in all α and β PFTs (Fig. 2, Supplementary Figs. 1–3)^11^. The architecture of this domain is responsible for the structural distortion of most of the PFTs. Notably, this conformational switch is often considered as the characteristics of the shape-shifting proteins which are utilized by many pathogenic systems to protect them from the invaders through allosteric communication between distant regions of the protein^12^. Corynebacterium diphtheria also utilizes the low pH driven refolding of Translocation-domain (T-domain) for the activation of membrane-competent W-state from membrane-incompetent W-state to instigate the membrane pore formation in host cells for the successful infection^13^. It is noteworthy that leishmania donovani parasite-derived surface active protein KMP-11 utilizes its N-terminal domain (1–33 AA) to induce pores in host membranes to establish infection^14, 15^. Interestingly, the sequence alignment results show that there is significant sequence similarity between KMP-11 and MPT63. It has been found that the pore-forming domains of these two proteins are conserved (Fig. 2, Supplementary Figs. 1, 4–7).

Toxins that show conformational switches very often contain a number of special chameleon sequences, which can adopt either alpha or beta-sheet depending upon the context^16^. Interestingly, chameleon sequences that switch between alpha-helix and strand contain stronger helix and/or beta when compared to average alpha-helices and beta-sheets containing proteins. The adoption of different conformations depends on the packing of the hydrophobic residues to create a binary pattern of the chameleon sequences. The hydrophobic residues packing is easily accommodated by a beta-strand that adopts native binary pattern of *phphph* (where *p* and *h* denote the polar and hydrophobic residues respectively) and an alpha-helix with *phhpph* pattern. Previous study with the Arc repressor has pointed out that the slight alteration in a single methyl group in mutants containing ambiguous binary pattern *phhhph* can result a conformational switch^17^. Our recent work suggested that MPT63 in MTB utilizes its chameleon sequences (these sequences also posses ambiguous binary patterns) to switch from native beta-sheet state to alpha-helical structure^18^. This alpha-helical conformation generates toxic oligomers at pH 5. These toxic oligomers create pores in macrophages and model membrane.

## Conformational switches in viral proteins

In addition to the bacterial and parasite systems, viral systems employ specific fusogenic proteins to invade the host membranes. Influenza hemagluttinin is an example of a viral protein that uses its ‘spring loaded’ metastable conformation in the presence of pH change trigger, which switches to its fusogenic form^19^. The fusion protein of influenza C which is known as HEF contains an esterase domain along with the receptor-binding domain and fusion domain. The esterase domain helps to hydrolyze the 9-O-acetyl-sialic acid receptor for the viral escape. Apart from the extra presence of the esterase module, HEF is considerably similar to HA of influenza A^20^.

On the other hand, during viral internalization process the HIV and SIV envelope glycoproteins carry out two crucial functions. They catalyze the fusion after making the membrane attachment. There occurs a cleavage in the envelope glycoprotein (gp160) by furin-like protease during the late stage of export pathway^21^. Although there is no strong attachment between two cleaved fragments (gp120 and gp 41), they remain associated^22^. There occurs a conformational change in gp140/gp41 due to their binding with CD4 receptor. This conformational changes lead to the alteration in immunogenicity, enhancement of proteolytic sensitivity of the gp120 moiety, and enhanced shedding^23^. This conformational change increases the binding affinity to the co-receptor in a co-operative manner^24^. Co-receptor attachment leads to fusion, by inducing gp120 dissociation, exposing fusion peptide, and gp41 refolding^25^.

**Fig. 3:**
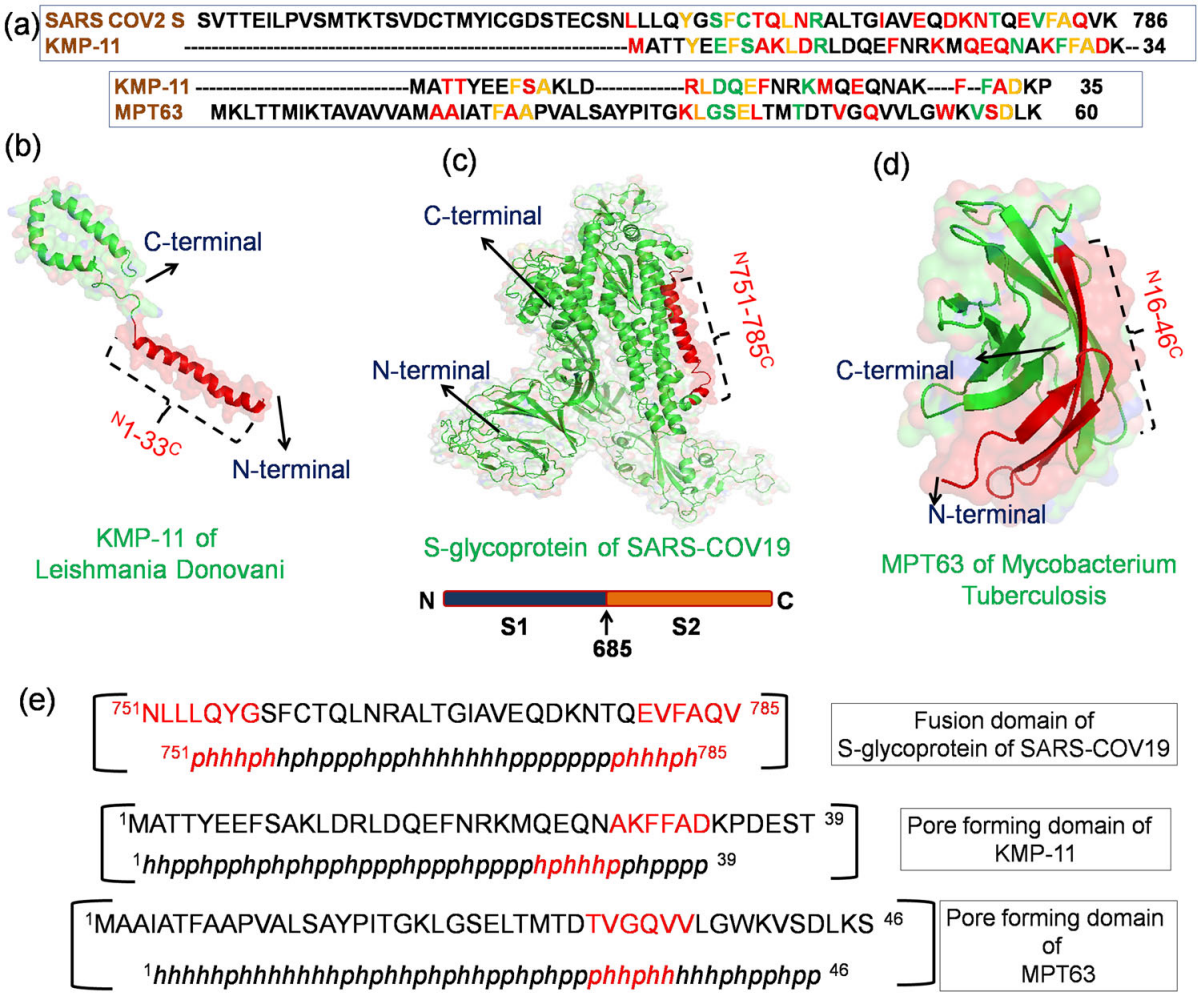
The special characteristics of the fusion domains of different pathogen-derived proteins. **a** The sequence similarities of KMP-11 (Leishmania Donovani parasite derived) with S-glycoprotein (QHD3416) (coronavirus derived) and MPT63 (Mycobacterium Tuberculosis derived) (1LMI) proteins have been determined using multiple sequence alignment. Here, the residues have been color marked according to their similarity. The yellow color indicates ‘exact’, red color denotes ‘conserved substitution’ and green color stands for ‘semi conserved substitution’. The model structures of **b** KMP-11, **c** S-glycoprotein and **d** MPT63 are shown. The common sequence motif (conserved domain) of these three proteins of different pathogen origins is color coded by red. These common motifs may be responsible for the conformational switch from their native inactive to active states. **e** The sequences of the pore-forming domain of different proteins have been arranged with respect to hydrophobicity and polarity. There exist two sequence ambiguities (*phhhph*) in Spike glycoprotein of COVID19 conserved domain. One remains at the starting and other one at the end. Interestingly, KMP-11 contains similar sequence pattern whereas, MPT63 contains ambiguous chameleon pattern (*phhphh*). Here, the amino acid sequences have been arranged on the basis of hydrophobicity and polarity of the residues and relative hydrophobicity and polarity parameters were calculated using Wimley–White interfacial hydrophobicity scale (WWIHS).

Very recently, the severe acute respiratory syndrome (SARS) coronavirus 2 (COVID-19) has emerged as a brutal global pandemic. To design a promising drug against COVID 19, we need to understand the procedure of its infection. The principal protein molecule responsible for establishing infection is the surface spike protein (S)^26^. This spike protein (S) consists of two segments: spike protein S1 (14–685 AA) and spike protein S2 (686–1273 AA). S1 attaches the virion to the cell membrane by interacting with the host receptor (ACE2) initiating the infection. Binding to human ACE2 and internalization of the virus into the endosomes of the host cell induces conformational changes in the S glycoprotein. The protein has three conformational states: pre-fusion native state, pre-hairpin intermediate state, and post fusion hairpin state. During viral and target cell membrane fusion, the coiled-coil regions (heptad repeats) assume a trimer-of-hairpins structure, positioning the fusion peptide in close proximity to the c-terminal region of the ectodomain. The formation of this structure appears to drive apposition and subsequent fusion of viral and target cell membranes. During its entry process, the S-protein (specifically S2 segment) also undergoes a conformational switch. Although, viral and bacterial systems utilize their own protein molecules for infection in host, a significant sequence similarity can be found between the PFTs and the viral fusion proteins. It is surprising to see that a common motif is present in all these protein molecules (Figs. 2 and  3). It is also interesting to note that the fusion domain of the spike protein of SARS COV2 contains a particular ambiguous binary pattern (*phhhph*) at the initial and final portion in the common sequence motif (Fig. 3). The hydrophobicity and polarity of the residues were determined using Wimley–White interfacial hydrophobicity scale (WWIHS)^27^. This nature of sequence suggested the involvement of these specific domains during the conformational state transition in presence of trigger factors. We can suggest that this particular segment is the decisive factor behind SARS COV2 fusion with host at the early stage of infection. The pore-forming domain of KMP-11 contains similar pattern (*phhhph*) as the S-protein. On the other hand, slightly different binary pattern (*phhphh*) exists in the pore-forming domain of MPT63 and this domain is already known as a chameleon stretch. We believe that these types of ambiguous binary pattern remain conserved in the common sequence region of different protein species of different origins.

## Conformational switch for new edge therapeutics development

While the conformational switch strategy has been utilized by the pathogenic systems for their survival inside host, researchers have attempted to utilize proteins conformational switch behavior to the medicinal and therapeutic purposes. To design new proteins that can switch conformation, protein designers have focused on the two key components of protein switches: the amino acid sequence must be compatible with the multiple target states and there must be a mechanism for perturbing the relative stability of these states. Recent reports present a general strategy to design pH-responsive protein conformational changes by precisely pre-organizing histidine residues in buried hydrogen-bond networks^28^. A number of groups are working on hydrogel for targeted drug delivery purpose. In this regard, the conformational switching from random coil to β-hairpin structures of selected MAX1 peptide was used^29^. The use of nanoparticles (NPs) may also be useful in different applications. Very recently, we have shown that MTB derived WT MPT63 protein undergoes beta to alpha-helical transition when it gets attached on NP surface. During this switch, there occurs a partial unfolding which leads to the loss of immunogenicity of the protein. Since the loss of immunogenicity may be detrimental toward the development of a nano-vaccine, we have artificially generated the conformational switch in the protein through a slight change in the sequence using protein engineering technique. Our engineered point mutant of MPT63 (W26F) showed appreciable immunogenicity while bound onto the NPs surface, and this strategy can be used to design and synthesize new generation nano-based vaccine formulation^30^. The specific sequence stretch which is responsible for the conformational switch of a protein needs to be probed not only for making protein drug formulation but also for designing novel small molecules to target specific protein domain. The study of crystal structure of many proteins presents multiple pockets where small molecule binds easily and shows the path of allostery development. This pathway of allostery development is not applicable for every case since many proteins do not contain any particular drug binding site. It is interesting to note that protein-shape shifting may populate in the high energy conformational state through the modulation of specific pockets (or cryptic pockets), which cannot be resolved through the crystal structure analysis^31^. These cryptic pockets can easily overlap with the allosteric network. Small molecule can bind to the active sites by allosteric way either through the activation or inhibition of the process resulting in the modulation of relative populations of different protein structures.

Decoding the particular sequence motif in this context may help in optimal drug design against pathogenic and viral infections including that of COVID 19. Due to the ever-changing behavior of the bacteria and the viruses, the widely accepted ‘one bug-one drug’ approach has been changing and the need for the development of broad-spectrum anti-virals targeting the viral entry process is increasing. A number of peptide inhibitors has been designed and used for viral entry inhibition. Previous reports suggested that the heptad repeat units (HR1 and HR2) in the S2 domain of SARS-CoV has been targeted to design potential peptide inhibitors^32^. A number of strategies like rational, intentional, structure-based, accidental, and brute force are available for the design and invention of new peptide entry inhibitors^33^. The peptide entry inhibitors can interfere with the fusion of cellular and viral membranes through the simultaneous alteration of the membrane-physical property and blocking the fusion protein interfaces as competitive inhibitors. In this context, the interfacial hydrophobicity of putative entry inhibitors can lead to the efficient discovery of novel, broad-spectrum viral entry inhibitors. Similar approaches can be used for the generation of broad-spectrum peptide entry inhibitors to target and inhibit the special sequence stretch (that contains the ambiguous binary pattern) responsible for conformational switch driven host membrane fusion process to mitigate the infections including SARS COV2.

## Conclusions

There exists a structural correlation between the functional states of different pathogen-derived proteins. Some pathogenic systems usually use their pore-forming proteins for their entry inside host. These pore-forming proteins are classified into different classes depending on their structures. Interestingly, few pathogenic secretory proteins utilize their conformational switch strategy for membrane pore formation. These types of protein molecules contain specific chameleon sequences which enable them to switch from their inactive to active states during the process of infection. Several viral systems use the fusion domains of their envelope proteins for viral entry. In this perspective, to our knowledge, we demonstrate for the first time a correlation between the pore-forming and fusion domains of proteins of different pathogenic origins. We have shown that there exists a specific ambiguous binary sequence pattern in the pathogen-derived protein sequences. These specific domains may remain conserved in multiple pore-forming proteins that utilize environment-sensitive conformational switch strategy. Finally, we have discussed how the conformational switch strategy can be utilized toward novel therapeutics developments.

## Supplementary information

Supplementary Information

## Data Availability

All software used in this paper is freely available on the web.
