# Supplementary material for: The bright and dark sides of protein conformational switches and the unifying forces of infections
Source: Commun Biol. 2020 Jul 15;3:382. doi: 10.1038/s42003-020-1115-x (PMC7363679; doi:10.1038/s42003-020-1115-x)
Supplement: Supplementary file 1 — Supplementary Information [file 42003_2020_1115_MOESM1_ESM.pdf]

Supplementary Information

Methods

The structures of the proteins have been retrieved from RCSB PDB (https://www.rcsb.org/) and the structures were visualized in pymol software. The sequence similarities between different protein sequences were determined by multiple sequence alignment clustal omega softwere (https://www.ebi.ac.uk/Tools/msa/clustalo/).

```
sp|Q36736|KMP11      --------------MATTYEEFSAKLD--------RLDQEFNRK--MQEQNAKF-FADKP      35
1LMI                 MKLTTMIKTAVAVVAMAAIATFAAPVALSAYPITGKLGSELTMTDTVGQVVLGWKVSDLK      60
                                     ::   *:* :         :*..*:. .  : :    : .:*

sp|Q36736|KMP11      DESTLSPEMR---EHYEK------FERMIKEHTEKFNKKMHEHSEHFKQKFAEL-LEQQK      85
1LMI                 SSTAVIPGYPVAGQVWEATATVNAIRGSVTPAVSQFNARTADG-INYRVLWQAAGPDTIS      119
                     ..::: *      : :*       :.  :.  ..:** :  :   :::  :     :  .

sp|Q36736|KMP11      AAQYPSK---------------------------------      92
1LMI                 GATIPQGEQSTGKIYFDVTGPSPTIVAMNNGMEDLLIWEP      159
                     .*  *.
```

Supplementary Figure 1 : Sequence similarity between KMP-11 and MPT63 as evaluated by multiple sequence alignment study. The sign ‘*’indicates ‘exact’, ‘:’denotes ‘conserved substitution’ and ‘.’stands for ‘semi conserved substitution’.

```
5JZH:A|PDBID|CHAIN|SEQUENCE      AEPVYPDQLRLFSLGQGVCGDKYRPVNREEAQSVKSNIVGMMGQWQISGLANGWVIMGPG    60
1LMI:A|PDBID|CHAIN|SEQUENCE      ------------------------------------------------------------    0

5JZH:A|PDBID|CHAIN|SEQUENCE      YNGEIKPGTASNTWCYPTNPVTGEIPTLSALDIPDGDEVDVQWRLVHDSANFIKPTSYLA    120
1LMI:A|PDBID|CHAIN|SEQUENCE      ------------------------------------------------------------    0

5JZH:A|PDBID|CHAIN|SEQUENCE      HYLGYAWVGGNHSQYVGEDMDVTRDGDGWVIRGNNDGGCDGYRCGDKTAIKVSNFAYNLD    180
1LMI:A|PDBID|CHAIN|SEQUENCE      ------------------------------------------------------SAY---    3
                                                                                         **

5JZH:A|PDBID|CHAIN|SEQUENCE      PDSFKHGDVTQSDRQLVKTVVGWAVNDSDTPQ---SGYDVTLRGDTATNWSKTNTYGLSE    237
1LMI:A|PDBID|CHAIN|SEQUENCE      PITGKLGSELTMTDTVGQVVLGWKVSDLKSSTAVIPGYPVA----GQVWEATATVNAIR    58
                                 * : * *.       : :.*:** *.* .:      ** *:     .  *. * * .  .

5JZH:A|PDBID|CHAIN|SEQUENCE      --------------KVTTKNKFKWPLVGETELSIEIAANQSWASQNGGSTTTSLSQSVRP    283
1LMI:A|PDBID|CHAIN|SEQUENCE      GSVTPAVSQFNARTADGINYRVLWQAAGPDTISGA-------------------------    93
                                                   : :. *  .*   :*

5JZH:A|PDBID|CHAIN|SEQUENCE      TVPARSKIPVKIELYKADISYPYEFKADVSYDLTLSGFLRWGGNAWYTHPDNRPNWNHTF    343
1LMI:A|PDBID|CHAIN|SEQUENCE      TIPQGEQSTGKIY-F--DVTGPS--PTIVAMNNGMEDLLIWEP-----------------    131
                                 *:*  .:   **  :  *:: *    :  *: :  :..:* *

5JZH:A|PDBID|CHAIN|SEQUENCE      VIGPYKDKASSIRYQWDKRYIPGEVKWWDWNWTIQQNGLSTMQNNLARVLRPVRAGITGD    403
1LMI:A|PDBID|CHAIN|SEQUENCE      ------------------------------------------------------------    131

5JZH:A|PDBID|CHAIN|SEQUENCE      FSAESQFAGNIEIGAPVPLAA  424
1LMI:A|PDBID|CHAIN|SEQUENCE      ---------------------  131
```

Supplementary Figure 2: Sequence similarity between MPT63 and Aerolysin as evaluated by multiple sequence alignment study. The sign '*'indicates 'exact', ':'denotes 'conserved substitution' and '.'stands for 'semi conserved substitution'.

```
1IAZ:A|PDBID|CHAIN|SEQUENCE      --------------SADVAGAVIDGASLSFDILKTVLEALGNVKRKI-AVGVDNESGKTW   45
1LMI:A|PDBID|CHAIN|SEQUENCE      SAYPITGKLGSELTMTDTVGQVVLGWKVS------------DLKSSTAVIPGYPVAGQVW   48
                                                :*..* *: * .:*            ::* .  .:     :*:.*

1IAZ:A|PDBID|CHAIN|SEQUENCE      TALNT---------------------------YFRSGTSDIVLPHKVPHGKALLYNGQKD   78
1LMI:A|PDBID|CHAIN|SEQUENCE      EATATVNAIRGSVTPAVSQFNARTADGINYRVLWQAAGPDTISGATIPQGE-------QS   101
                                  *  *                            :::.  * :   .:*:*:        :.

1IAZ:A|PDBID|CHAIN|SEQUENCE      RGPVATGAVGVLAYL--MSDGNTLAVLFSVPYDYNWYSNWWNVRIYKGKRRADQRMYEEL   136
1LMI:A|PDBID|CHAIN|SEQUENCE      TGKIYFDVTGPSPTIVAMNNGMEDLLI--------WEP----------------------   131
                                  * :  ...*    :  *.:*    ::        *

1IAZ:A|PDBID|CHAIN|SEQUENCE      YYNLSPFRGDNGWHTRNLGYGLKSRGFMNSSGHAILEIHVSKA   179
1LMI:A|PDBID|CHAIN|SEQUENCE      -------------------------------------------   131
```

Supplementary Figure 3 : Sequence similarity between MPT63 and Equinatoxin-II as evaluated by multiple sequence alignment study. The sign ‘*’indicates ‘exact’, ‘:’denotes ‘conserved substitution’ and ‘.’stands for ‘semi conserved substitution’.

```
sp|Q36736.1|KM11_LEIDO          ------------------------------------------------------------   0
5JZH:A|PDBID|CHAIN|SEQUENCE     AEPVYPDQLRLFSLGQGVCGDKYRPVNREEAQSVKSNIVGMMGQWQISGLANGWVIMGPG   60

sp|Q36736.1|KM11_LEIDO          ------------------------------------------------------------   0
5JZH:A|PDBID|CHAIN|SEQUENCE     YNGEIKPGTASNTWCYPTNPVTGEIPTLSALDIPDGDEVDVQWRLVHDSANFIKPTSYLA   120

sp|Q36736.1|KM11_LEIDO          -----------------------------------------------MATTYEEFSAKLD   13
5JZH:A|PDBID|CHAIN|SEQUENCE     HYLGYAWVGGNHSQYVGEDMDVTRDGDGWVIRGNNDGGCDGYRCGDKTAIKVSNFAYNLD   180
                                                                                * . .:*: :**

sp|Q36736.1|KM11_LEIDO          RLDQEF------NRKMQEQNAKFFADKPDE------STLSPEMREH------YEKFERMI   55
5JZH:A|PDBID|CHAIN|SEQUENCE     PDSFKHGDVTQSDRQLVKTVVGWAVNDSDTPQSGYDVTLRGDTATNWSKTNTYGLSEKVT   240
                                  . :.      :*:: :  . : .:. *        **  :   :      *   *::

sp|Q36736.1|KM11_LEIDO          KEHTEKFNKK------------------------------------------MHEHSEHF   73
5JZH:A|PDBID|CHAIN|SEQUENCE     TK--NKFKWPLVGETELSIEIAANQSWASQNGGSTTTSLSQSVRPTVPARSKIPVKIELY   298
                                .:  :**:                                            :  : * :

sp|Q36736.1|KM11_LEIDO          KQK----------------FAELLEQQKAAQY----------------------------   89
5JZH:A|PDBID|CHAIN|SEQUENCE     KADISYPYEFKADVSYDLTLSGFLRWGGNAWYTHPDNRPNWNHTFVIGPYKDKASSIRYQ   358
                                * .                ::  :*.    * *

sp|Q36736.1|KM11_LEIDO          ------PSK---------------------------------------------------   92
5JZH:A|PDBID|CHAIN|SEQUENCE     WDKRYIPGEVKWWDWNWTIQQNGLSTMQNNLARVLRPVRAGITGDFSAESQFAGNIEIGA   418
                                      *.:

sp|Q36736.1|KM11_LEIDO          ------ 92
5JZH:A|PDBID|CHAIN|SEQUENCE     PVPLAA 424
```

Supplementary Figure 4 : Sequence similarity between KMP-11 and aerolysin (5JZH) as evaluated by multiple sequence alignment study. The sign '*'indicates 'exact', ':'denotes 'conserved substitution' and '.'stands for 'semi conserved substitution'.

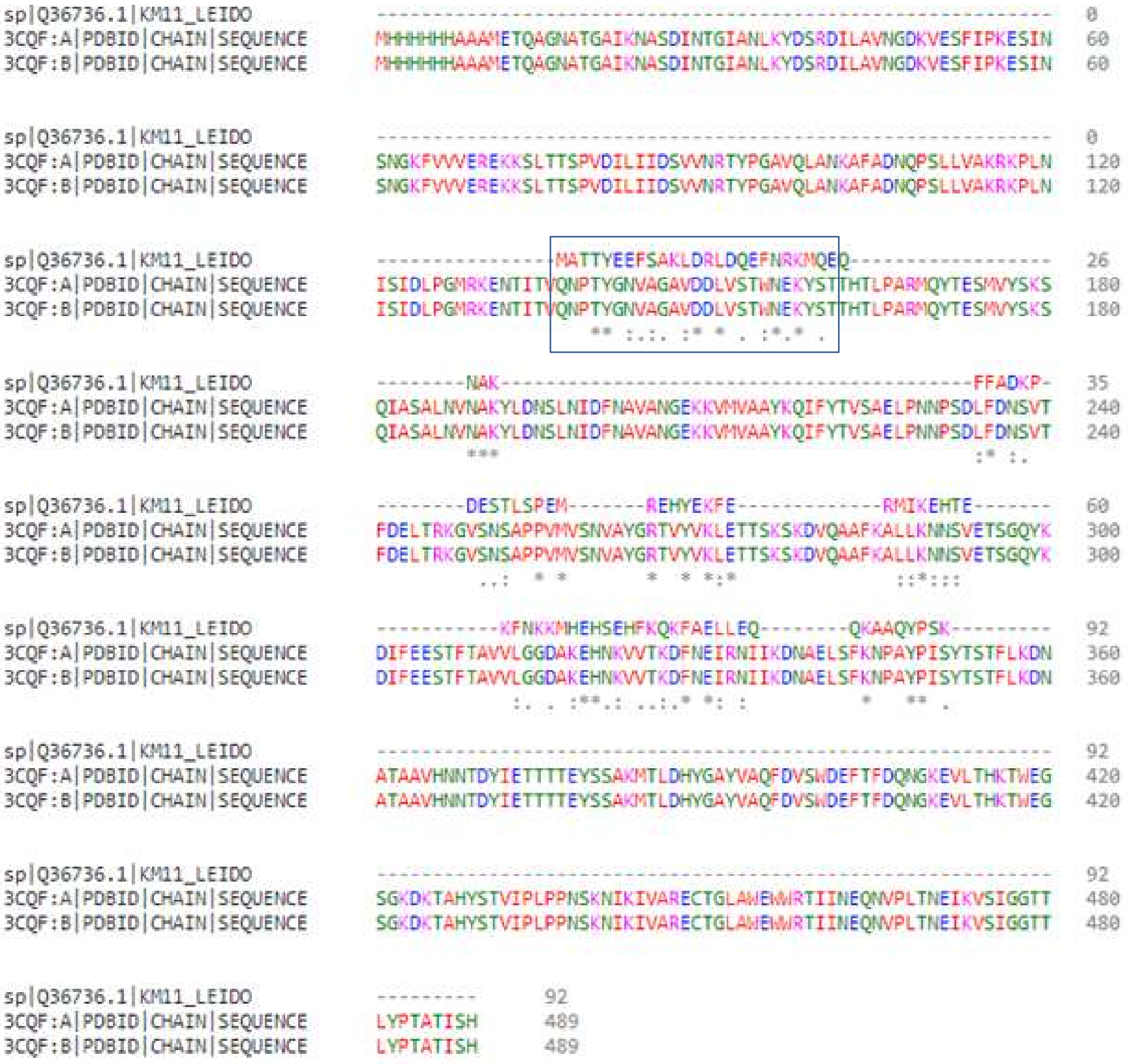

Supplementary Figure 5 : Sequence similarity between KMP-11 and Anthrolysin o (3CQF) as evaluated by multiple sequence alignment study. The sign ‘*’indicates ‘exact’, ‘:’denotes ‘conserved substitution’ and ‘.’stands for ‘semi conserved substitution’.

```
sp|Q36736.1|KM11_LEIDO          ----------MATTYEEFSAKLDRLD---QE-----------------------------   18
1IAZ:A|PDBID|CHAIN|SEQUENCE     SADVAGAVIDGASLSFDILKTVLEALGNVKRKIAVGVDNESGKTWTALNTYFRSGTSDIV   60
1IAZ:B|PDBID|CHAIN|SEQUENCE     SADVAGAVIDGASLSFDILKTVLEALGNVKRKIAVGVDNESGKTWTALNTYFRSGTSDIV   60
                                           : ::: :.: *: *.    ::

sp|Q36736.1|KM11_LEIDO          FNRKMQEQNAKFFADKPDESTLSP------------------EM--REHYEKFERMIKEH   58
1IAZ:A|PDBID|CHAIN|SEQUENCE     LPHKVPHGKALLYNGQKDRGPVATGAVGVLAYLMSDGNTLAVLFSVPYDYNWYSNWWNV-   119
1IAZ:B|PDBID|CHAIN|SEQUENCE     LPHKVPHGKALLYNGQKDRGPVATGAVGVLAYLMSDGNTLAVLFSVPYDYNWYSNWWNV-   119
                                : :*: . :* :: .: *.. ::                 :    .*: :..  :

sp|Q36736.1|KM11_LEIDO          TEKFNKKMHEHSEHFKQKFAELLEQQKAAQYPSK--------------------------   92
1IAZ:A|PDBID|CHAIN|SEQUENCE     -RIYKGKRRADQRMYEELYYNLSPFRGDNGWHTRNLGYGLKSRGFMNSSGHAILEIHVSK   178
1IAZ:B|PDBID|CHAIN|SEQUENCE     -RIYKGKRRADQRMYEELYYNLSPFRGDNGWHTRNLGYGLKSRGFMNSSGHAILEIHVSK   178
                                 . :: * : ... ::: : :*   :    : ::

sp|Q36736.1|KM11_LEIDO          -      92
1IAZ:A|PDBID|CHAIN|SEQUENCE     A      179
1IAZ:B|PDBID|CHAIN|SEQUENCE     A      179
```

Supplementary Figure 6: Sequence similarity between KMP-11 and Equinatoxin-II as evaluated by multiple sequence alignment study.

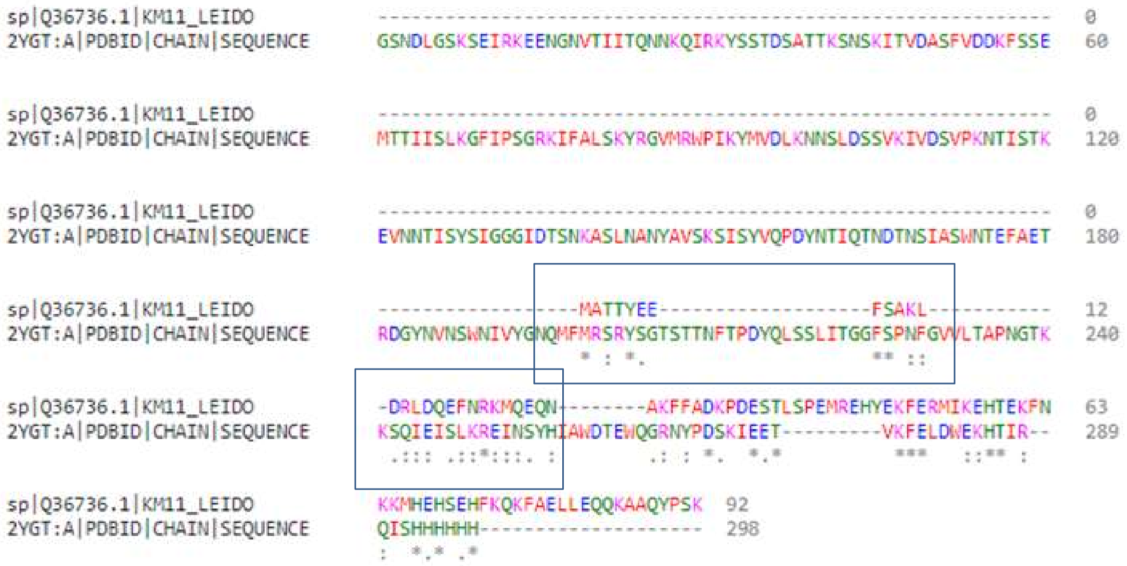

Supplementary Figure 7: Sequence similarity between KMP-11 and delta toxin as evaluated by multiple sequence alignment study. The sign '*'indicates 'exact', ':'denotes 'conserved substitution' and '.'stands for 'semi conserved substitution'.
